# Best evidence topic: Should patients with asymptomatic choledocholithiasis be treated differently from those with symptomatic or complicated disease?

**DOI:** 10.1016/j.amsu.2020.12.048

**Published:** 2021-01-13

**Authors:** Hossam Nawara, Rashid Ibrahim, Sabry Abounozha, Awad Alawad

**Affiliations:** a Derriford Hospital, University Hospital Plymouth NHS Trust, Plymouth, UK; b Northumbria Healthcare NHS Foundation Trust, Northumbria, UK; c University Hospital of Wales, Cardiff, UK

**Keywords:** Choledocholithiasis, CBD stones, Bile duct stones, ERCP, Asymptomatic

## Abstract

Choledocholithiasis is a common finding in clinical practice, with presentation varying from asymptomatic to life-threatening complications. In symptomatic patients, there is no doubt that treatment to clear the bile duct is indicated, but there is still a debate regarding the treatment of patients with silent common bile duct stones (CBDS). The question addressed by this best evidence topic is whether patients with asymptomatic CBDS should be managed in the same way as patients with symptoms or complications. The search strategy yielded 609 articles, from which 8 articles found to be relevant to this topic. We also summarised the most notable societal guidelines recommendations, regarding this topic. We tabulated the article title, author, year, country, study type, outcomes, results, and comments. We concluded that patients with asymptomatic CBD stones should be offered endoscopic treatment If they are fit, after discussion of the potential risks and benefits of both options of conservative and interventional treatment with the patients.

## 1 Introduction

This BET was designed using a framework outlined by the International Journal of Surgery [1]. This format was used because a preliminary literature search suggested that the available evidence is of insufficient quality to perform a meaningful meta-analysis. A BET provides evidence-based answers to common clinical questions, using a systematic approach of reviewing the literature.

Choledocholithiasis is a common complication of gallstone disease that presents to clinicians in a variety of modes ranging from being incidentally discovered silent CBDS to life-threatening complications such as pancreatitis. There is a general consensus that symptomatic patients with CBDS should be offered treatment which in most clinical settings will be ERCP. However, the treatment of silent CBDS remains a matter of debate. This can be explained by the paucity of evidence regarding the natural history of CBD stones. In this topic we attempted to answer the question of whether silent CBD stones should be managed differently from symptomatic disease, by looking at data on the natural history of silent CBD stones, and the outcome of their management in asymptomatic patients.

## 2 Clinical scenario

A 70 years old female patient, undergoing investigations for change in bowel habit with a Computed Tomography (CT) colonography. The scan didn't reveal any colonic abnormality, but showed a CBD stone measuring 7 mm. She denied having any symptoms related to gallstone disease, and her Liver function tests are normal. She had undergone laparoscopic cholecystectomy 2 years earlier. You wonder whether she should be referred for ERCP or managed conservatively.

## 3 Three part question

(1) Should patients with asymptomatic CBD stones [2] offered the same treatment as symptomatic patients or [3] Should they be managed conservatively.

## 4 Search strategy

Medline search from 1978 to 2020, limited to English language articles.

### 4.1 Search phrase

(((Treatment*[Title/Abstract]) OR (Natural History [Title/Abstract) OR (Management [Title/Abstract]) OR (ERCP [Title/Abstract]) OR (Endoscop*[Title/Abstract])) AND ((Choledocholithiasis [Title/Abstract]) OR (CBDs [Title/Abstract]) OR (CBD [Title/Abstract]) OR (bile duct stone*[Title/Abstract]) OR (bile duct calcul*[Title/Abstract])) AND ((symptomatic) OR (asymptomatic) OR (Silent))) AND (English [Language])

### 4.2 Inclusion criteria

• Studies on the natural history of CBD stones.
• Studies comparing treatment of CBDS in symptomatic and symptomatic patients.
• Review articles and guideline papers on the management of CBDS.

### 4.3 Exclusion criteria

• Studies comparing different treatment modes (Endoscopic, Surgical)
• Studies on paediatric population.
• Irrelevant articles.

## 5 Search outcome

• The search outcome yielded 609 results, which were filtered one by one by going through the title and the summary in order to look for the relevant articles. This yielded 23 papers which were further filtered by reading through the main paper. 3 duplicate articles were excluded and 12 articles were found relevant. The rest were irrelevant to the topic and were excluded.
• 8 study articles were found relevant to the topic, summarised in Table 1.

**Table 1:** Summary of Relevant studies.

Article	Author, Date, Country, Type of study	Patient groups	Outcomes	Key results	Comments
Natural history of asymptomatic bile duct stones and association of endoscopic treatment with clinical outcomes	Hakuta et al. [2], 2019, Japan. Retrospective longitudinal cohort study	191 patients (114 patients in the wait and see group, and 77 patients in the intervention group.	Biliary complications in both groups, Post ERCP complications in intervention group, and Asymptomatic disappearance of stones in wait-and-see group. Median Follow-up: 3.2 years in Wait and see group, and 1.9 years in the intervention group.	•Cumulative incidence of Biliary complications in wait-and-see group: 6.1% at 1 year, 11% at 3 years, and 17% at 5 years. •Asymptomatic disappearance of stones in 22 patients (19%). •Procedure-related complications: 25 patients (32%), of which 4 (5.2%) with severe pancreatitis.	
A Prospective Study of Common Bile Duct Calculi in Patients Undergoing Laparoscopic Cholecystectomy Natural History of Choledocholithiasis Revisited	Collins et al. [3], 2004, Ireland Retrospective study	962 patients had Intraoperative cholangiography (IOC) during Laparoscopic/open cholecystectomy. 34 patients found to have ductal stones. Follow-up of these patients was done.	Persistent CBD stones, or spontaneous duct clearance at 6-weeks follow-up.	•One third of patients (12/34) had spontaneous duct clearance at 6-weeks follow-up post cholecystectomy	Small number of patients, short follow-up period
Routine vs “on demand” postoperative ERCP for small bile duct calculi detected at intraoperative cholangiography Clinical evaluation and cost analysis	Ammori et al. [4], 1999, UK Prospective study	705 patients had IOC during Laparoscopic cholecystectomy. 70 patients found to have ductal stones.	44 out of 70 patients had large calculi at laparoscopic cholecystectomy (LC), and underwent Laparoscopic CBD exploration. The remaining patients were assigned to either postoperative ERCP (Group A, n = 8), or observation (Group B, n = 14). Follow-up: 18 months to 3 years	• No complications developed in Group B during the follow-up period (median = 18 months), but 4 patients became symptomatic and needed ERCP.	Selection bias (All patients with stones ≥ 5 mm were excluded, patients individually assigned to both groups. Small sample size
Natural course vs interventions to clear common bile duct stones: data from the Swedish Registry for Gallstone Surgery and Endoscopic Retrograde Cholangiopancreatography (GallRiks)	Möller et al. [5], 2014, Sweden. Retrospective cohort analysis.	3828 patients with CBD stones discovered on IOC out of 38864 cholecystectomies.	Complication rates and/or incomplete clearance and need for intervention (Unfavourable Outcomes). Follow-up: 6 months	• Risk of unfavourable outcome higher in patients who had no intraoperative measures (25.3%) than in whom any measure was taken to clear the duct (12.7%)	Large sample size. The study included post-ERCP complications as part of Unfavourable outcomes of not treating CBDS at surgery.
Endoscopic treatment for choledocholithiasis in asymptomatic patients	Xiao-dan Xu et al. [6], 2019, China. Prospective comparative study.	327 consecutive patients with CBD stones (53 in the asymptomatic group and 274 in the symptomatic group) underwent ERCP to remove CBDs.	ERCP-related complications in both groups: Post ERCP Pancreatitis (PEP), Cholangitis, Perforation, Bleeding.	• Total of 46 patients (14.1%) had post ERCP complications. • Overall complication rate is higher in asymptomatic than symptomatic group (26.4% vs 11.7%, P < 0.01). • Higher incidence of PEP in asymptomatic than symptomatic group (20.8% vs 6.9%, P < 0.01).	
Post-endoscopic retrograde cholangiopancreatography pancreatitis in patients with asymptomatic common bile duct stones	Saito et al. [7], 2019, China. Retrospective study.	1113 patients with choledocholithiasis were included (949 symptomatic, and 164 asymptomatic)	Incidence of PEP in both groups.	Incidence of PEP was significantly higher in the asymptomatic than the symptomatic group (3% vs 14.6%, P < 0.001, odds ratio = 56)	
Comparison of Outcomes and Complications of Endoscopic Common Bile Duct Stone Removal Between Asymptomatic and Symptomatic Patients	Kim et al. [8], 2015, USA. Retrospective study	568 patients who underwent ERCP treatment for CBD stones (symptomatic in 536, and asymptomatic in 32)	Outcomes on complications of ERCP between the two groups.	Success rate of CBD stone removal is comparable in both groups. PEP is higher in the asymptomatic group (12.5 vs 3.9%, p = 0.045)	
Common Bile Duct Dilatation with Stones Indicates Requirement for Early Drainage in Patients with or Without Cholangitis	Yamashita et al. [9], 2013, Japan. Retrospective study	Clinical characteristics of 101 patients (66 symptomatic, and 35 asymptomatic) that underwent ERCP and drainage as Emergent (37 patients) or scheduled procedure.	Risk factors for the development of cholangitis requiring emergent drainage in patient with silent CBD stones.	Dilated CBD (>10 mm) was the only risk factor for cholangitis requiring emergent ERCP in patients with asymptomatic CBD stones.	Small sample size
• 4 relevant guidelines articles were found and summarised in Table 2.

**Table 2:** Summary of National Guidelines on the management of asymptomatic Choledocholithiasis.

Institute	Recommendation
The National Institute for Health and Care Excellence Guidelines (NICE) [10] 2014	Offer Bile duct clearance and cholecystectomy to people with symptomatic or asymptomatic CBD stones.
European Society of Gastrointestinal Endoscopy (ESGE) [11] 2019	Recommends offering stone extraction to all patients with CBD stones, symptomatic or not, who are fit for the procedure. Strong recommendation, low quality evidence.
British Society of Gastroenterology (BSG) 2017 [12]	In line with NICE guidelines, stone extraction should be offered to all patients with CBD stones. It should be noted that there are no controlled studies examining natural history of asymptomatic CBD stones. Recommends further research in this area.
European Association for the Study of the Liver (EASL) [13] 2016	Symptomatic Choledocholithiasis should be treated. No clear guidance on Asymptomatic choledocholithiasis.

## 6 Discussion

Choledocholithiasis is one of the common complications of gallstone disease, defined as the presence of stones within the common bile duct. This presence can be secondary to passing of a stone from the gallbladder into the bile duct, which is the most common type, or less commonly be formed de-novo inside the CBD. The presentation of CBD stones can vary from being asymptomatic, to life-threatening complications such as jaundice, cholangitis, pancreatitis.

There is a wide agreement among clinicians that patients with symptoms or complications from CBD stones should be offered treatment to clear the duct. The type of treatment will be influenced by several factors, including the timing when the diagnosis is made e.g. preoperative, during cholecystectomy, or postoperative, anatomical factors, e.g. previous gastric surgery, or congenital anomalies, and local expertise. Whatever the modality, the goal is to clear the bile duct of all stones, and provide adequate biliary drainage. This is strongly recommended by the most notable national guidelines [10,11,14].

This consensus turns into debate and disagreement in case of silent CBD stones, that may be discovered during cholecystectomy by using IOUS or IOC, or found on abdominal imaging done for other reasons. Although some of the national guidelines recommend offering duct clearance to asymptomatic patients if they are fit for the procedure, the evidence for this is of low quality, and there is still no consensus on managing these patients. These guidelines also agree that further research on the natural history of CBD stones is still needed. Surgeons and gastroenterologists managing these patients, are often faced with an important question: Does the benefit of having ERCP and duct clearance outweigh the risks associated with the procedure?

In order to answer this question, we need to have knowledge of the natural history of asymptomatic CBD stones, and what are the consequences If they are left alone, and the benefits and risks of ERCP in these patients.

There is paucity of data on the natural history of asymptomatic CBD stones. There are few retrospective cohort studies exploring the outcome of patients with silent CBD stones discovered during cholecystectomy using IOC, and even fewer studies looking at those that were discovered during imaging for other reasons.

One of these studies was conducted by Collins et al. [3] on 34 patients diagnosed with CBD stones discovered during laparoscopic cholecystectomy by routine IOC which was done for all patients with or without evidence of choledocholithiasis. When found, a *trans*-cystic catheter was left in place for 6 weeks. It was found that, after 6 weeks of follow-up, one third of these patients had asymptomatic spontaneous duct clearance, while the remaining had persistent filling defects on cholangiogram and underwent ERCP. The author concluded that It’s reasonable to manage the asymptomatic patients conservatively in the short term. Another study by Ammori et al. found that only 4 out of 14 patients with small CBD stones (<5 mm) became symptomatic during follow-up and needed ERCP [4]. They concluded that patients with asymptomatic small CBD stones can be managed expectantly, and only treated when they develop symptoms or complications.

In a recent retrospective cohort study by Hakuta et al. [2] in 2019, comparison between expectant and endoscopic treatment of asymptomatic CBD stones, showed cumulative incidence of complications in the expectant group to be 6.1%, 11%, and 17% at 1,3- and 5-years follow-up respectively, while the complication rate in the intervention group was 32% including 4 patients with severe pancreatitis. These figures imply that the natural history of silent CBD stones may favour wait-and-see management approach as an alternative to endoscopic treatment in asymptomatic patients.

On the other hand, data from the Swedish registry on 3828 patients that had cholecystectomy with CBD stones found on IOC, showed unfavourable outcome in 25.3%, which was significantly high, compared to 12.7% risk in those patients who had and form of treatment to clear the duct during surgery [5]. However, it's important to note that the unfavourable outcomes rate in this study also included post ERCP complications for patients that became symptomatic after surgery and had to have endoscopic treatment. We can argue that in the last scenario, these post intervention complications should be taken out of the equation, or even be included in the outcomes from intervention rather than wait-and-see group, which can change the results. This study also showed that there is a statistically significant association between increased CBD stone size and increased rate of symptomatic patients.

In the study by Yamashita et al. [9], multivariate analysis showed that CBD dilatation (>10 mm) was the only factor associated with increased risk of cholangitis requiring emergent endoscopic biliary drainage in both symptomatic and asymptomatic patients. Hence, they concluded that all patients; symptomatic and asymptomatic with CBD stones and dilated CBD (>10 mm) should undergo endoscopic treatment.

The aforementioned studies attempted to explore the natural history of silent CBD stones and If there was a difference in the outcome between interventional and wait-and-see treatment. What about the outcome from endoscopic treatment in these two groups? Is there a difference? Our search found three studies [[6], [7], [8]] (Xu et al. Saito et al., and Kim et al.) with a total of 2030 patients (253 asymptomatic and 1777 symptomatic patients) that compared ERCP treatment in symptomatic and asymptomatic groups. The main focus of these studies is to compare the risk of post-ERCP complications and particularly PEP in both groups. All of these studies showed higher risk of pancreatitis after ERCP for the asymptomatic than the symptomatic group. This higher incidence is suggested to be secondary to nondilated CBD, with small ampullary orifice, and prolonged cannulation time in asymptomatic group.

Measures to reduce the incidence of PEP include the use of rectal non-steroidal anti-inflammatory drugs immediately before or after ERCP, and prophylactic pancreatic stenting are advised in various guidelines [15,16]. These measures may be of particular importance in asymptomatic patients with higher risk of PEP.

Another crucial factor in reaching a decision on treatment of these patients, is considering their thoughts and opinions. Asymptomatic patients usually have higher expectations, and may be less tolerant to complications, as the procedure in their case is essentially prophylactic. After giving them all the necessary information, including the increased risk of endoscopic treatment, as well as the consequences of wait-and-see management, these discussions should be clearly documented in the patients’ notes. This becomes especially important in an increasingly litigious society.

## 7 Clinical bottom line

The current guidelines still recommend offering treatment to clear the CBD in asymptomatic patients, however the evidence for this is of low quality. There are data to suggest that wait-and-see can be a viable option, especially in high risk, or short life expectancy patients. Informed consent and discussion with patients about the potential benefits and potential higher risks is recommended in order to reach a management decision. When performed for asymptomatic patients, ERCP should be done by experienced endoscopists, and PEP preventative measures may be considered.

This study is limited by the paucity of studies on the natural history of CBDS, and the absence of controlled studies comparing interventional and conservative management of asymptomatic patients with CBDS. Further research on the natural history of CBD stones and controlled studies comparing various approaches to management of these patients is still required to reach a high-quality evidence.

## Ethical approval

Not Applicable.

## Source of funding

None.

## Author contribution

HN: Conducted the literature search and wrote the paper.

RI: Assisted in the literature search and writing of paper.

SA: Assisted in writing of paper.

AA: Assisted in the literature search, editing of writing.
